# Actinomycete-Derived Pigments: A Path Toward Sustainable Industrial Colorants

**DOI:** 10.3390/md23010039

**Published:** 2025-01-13

**Authors:** Blanca Hey Díez, Cristiana A. V. Torres, Susana P. Gaudêncio

**Affiliations:** 1 Associate Laboratory i4HB, Institute for Health and Bioeconomy, NOVA Faculty of Sciences and Technology, NOVA University of Lisbon, Campus Caparica, 2829-516 Caparica, Portugal; b.diez@campus.fct.unl.pt (B.H.D.); c.torres@fct.unl.pt (C.A.V.T.); 2 UCIBIO, Applied Molecular Biosciences Unit, Department of Chemistry, NOVA Faculty of Sciences and Technology, NOVA University of Lisbon, Campus Caparica, 2829-516 Caparica, Portugal

**Keywords:** actinobacteria, natural products, secondary metabolites, pigments, blue biotechnology, circular economy, sustainability, cosmetics, pharmaceutical, food, textile industries

## Abstract

Pigment production has a substantial negative impact on the environment, since mining for natural pigments causes ecosystem degradation, while synthetic pigments, derived from petrochemicals, generate toxic by-products that accumulate and persist in aquatic systems due to their resistance to biodegradation. Despite these challenges, pigments remain essential across numerous industries, including the cosmetic, textile, food, automotive, paints and coatings, plastics, and packaging industries. In response to growing consumer demand for sustainable options, there is increasing interest in eco-friendly alternatives, particularly bio-based pigments derived from algae, fungi, and actinomycetes. This shift is largely driven by consumer demand for sustainable options. For bio-pigments, actinomycetes, particularly from the *Streptomyces* genus, have emerged as a promising green source, aligning with global sustainability goals due to their renewability and biodegradability. Scale-up of production and yield optimization challenges have been circumvented with the aid of biotechnology advancements, including genetic engineering and innovative fermentation and extraction methods, which have enhanced these bio-pigments’ viability and cost-competitiveness. Actinomycete-derived pigments have successfully transitioned from laboratory research to commercialization, showcasing their potential as sustainable and eco-friendly alternatives to synthetic dyes. With the global pigment market valued at approximately USD 24.28 billion in 2023, which is projected to reach USD 36.58 billion by 2030, the economic potential for actinomycete pigments is extensive. This review explores the environmental advantages of actinomycete pigments, their role in modern industry, and the regulatory and commercialization challenges they face, highlighting the importance of these pigments as promising solutions to reduce our reliance on conventional toxic pigments. The successful commercialization of actinomycete pigments can drive an industry-wide transition to environmentally responsible alternatives, offering substantial benefits for human health, safety, and environmental sustainability.

## 1. Introduction

Pigments have been an integral part of human culture and artistic expression for thousands of years, beginning with prehistoric cave paintings dating back approximately 40,000 years [1]. Early humans utilized natural earth pigments like ochre, charcoal, and manganese oxide, ground from minerals and mixed with binders like animal fat or water [2,3]. These pigments were vital for depicting animals, human figures, and abstract symbols, reflecting their significance in early communication and spiritual practices. In ancient Egypt, pigments became more refined and were used not only for art but also for decoration and burial items. Egyptian Blue, the first synthetic pigment created around 2500 B.C., was made from silica, lime, copper, and alkali [4]. This blue pigment adorned tombs and artifacts, while lapis lazuli was ground into the costly ultramarine pigment for important religious artworks [5,6]. During the medieval and Renaissance periods, pigments gained associations with status and wealth. Notable pigments included lead white, gypsum, azurite, lazurite, indigo, malachite, vermilion, red lead, lead tin yellow (I), goethite, carbon, and iron gall ink [7]. The advent of synthetic pigments in the 18th and 19th centuries transformed art and industry. Prussian blue, discovered accidentally in 1704, became popular for its deep color and stability [8]. The Industrial Revolution further revolutionized pigment production, enabling mass manufacturing and making colors widely accessible [9,10]. This democratization of color allowed for the utilization of a broader palette, influencing textiles, painting, construction, and other industries.

**Figure 1:**
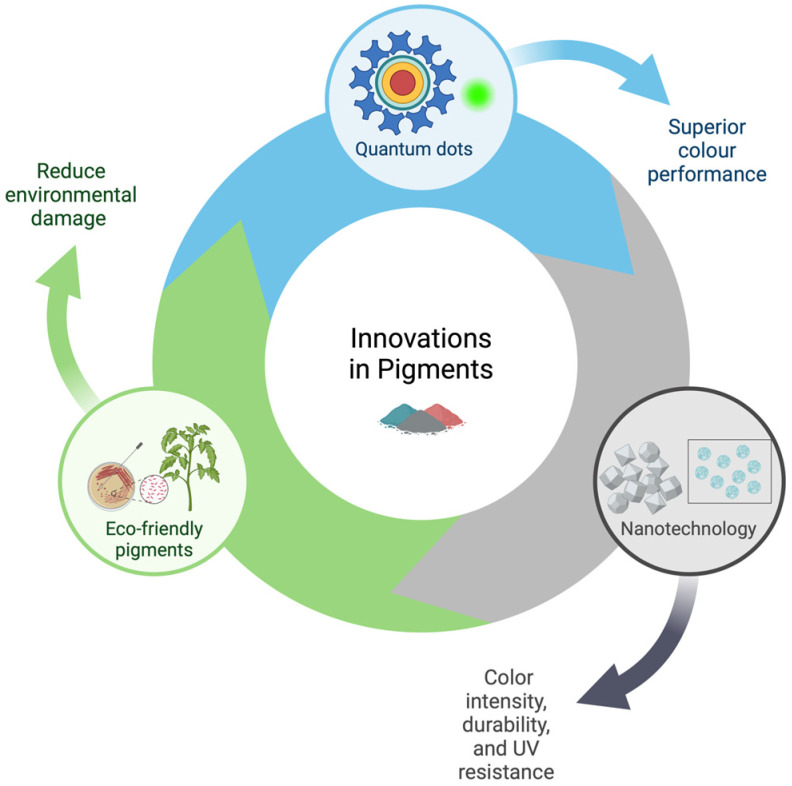
New trends in pigment technology. Created in https://BioRender.com.

Recent advancements in pigment technology have introduced significant innovations, such as quantum dots, nanotechnology, and eco-friendly pigments (Figure 1). Quantum dots, semiconductor nanoparticles, can emit colors when exposed to light, offering superior color performance in displays, coatings, art, and medical materials [11,12,13]. Nanotechnology has enabled the development of nanopigments, which enhance color intensity, durability, and UV resistance, making them valuable in industries like automotive paints, where they improve heat dissipation and self-cleaning capabilities [14,15]. Vantablack^®^ (Surrey NanoSystems Limited, East Sussex, UK), the “super-black” or the “darkest black”, made of carbon nanotubes, absorbs nearly all visible light, creating stunning visual effects. Additionally, plant-based pigments like turmeric and beetroot are being refined for use in art and cosmetics, alongside new biodegradable synthetic pigments [16]. There is also a focus on sustainable, eco-friendly pigments derived from natural sources, which can significantly reduce environmental impacts. Biodegradable and recycled pigments are gaining attention, aiming to replace traditional pigments while promoting a transition to a circular economy.

Actinomycetes, particularly the genus *Streptomyces*, have emerged as a significant source of natural pigments [17]. These Gram-positive bacteria produce a variety of pigments, such as melanin, prodigiosin, and actinorhodin, which exhibit diverse colors and valuable bioactive properties [18,19,20]. Actinomycete-derived pigments are appealing for their potential applications in food, cosmetics, textiles, and pharmaceuticals, combining aesthetic and functional benefits. Their sustainability is a key advantage, as these bacteria can be cultivated on renewable substrates and are biodegradable, in contrast to synthetic pigments often derived from petrochemicals [17].

The environmental impact of pigment production is substantial, with both natural and synthetic pigments contributing to ecological degradation. The shift towards sustainable alternatives, such as bio-based pigments and non-toxic substitutes, is reshaping the market in response to regulatory pressures and consumer preferences for eco-friendly products. Actinomycetes represent a promising avenue for natural, renewable, and biodegradable pigments that align with the growing demand for sustainable practices in various industries [17]. This review consolidates the literature on actinomycete-derived pigments over the past decade, offering the authors’ perspectives and success stories while emphasizing their significance in promoting sustainable pigment production. It highlights their potential in reducing the dependence on harmful synthetic dyes, fostering advancements in environmentally friendly and health-conscious alternatives.

## 2. Pigments in Modern Industries and Market Size

Pigments are extensively used in a variety of industries beyond traditional art, where their role is critical for adding color and improving the aesthetic appeal of products.

As of 2023, the global pigment market was valued at around USD 24.28 billion, with projections suggesting that it will grow to USD 36.58 billion by 2030. The market is expected to grow at a compound annual growth rate (CAGR) of approximately 6.03% during the forecast period from 2024 to 2030. Another estimate places the market at USD 29.45 billion in 2023, with a forecasted value of USD 38.76 billion by 2032, growing at a more moderate CAGR of 3.10% (https://www.maximizemarketresearch.com/market-report/global-pigments-market/15119/; https://www.precisionbusinessinsights.com/market-reports/pigments-market; accessed on 28 November 2024).

The market is experiencing a shift towards eco-friendly and organic pigments, reflecting a broader trend towards sustainability, non-toxicity, and the use of natural pigments in various industries, gaining momentum due to consumer demand for environmentally friendly products and tighter environmental regulations [21,22,23]. Pigments now play a role not just in aesthetics, but also in enhancing the performance and sustainability of products across many sectors. Due to the rising demand for natural and sustainable beauty products, there has been a shift toward using pigments derived from natural sources [24]. The textile industry is embracing digital pigment printing techniques, allowing for more complex designs and reduced water usage. Pigments play a crucial role in enhancing the visual appeal of foods and beverages, where color is closely tied to perceptions of taste and freshness, making them vital for consumer attraction. Beyond food applications, pigments are widely used in plastics for packaging, consumer goods, and industrial products, offering not only vibrant color and opacity but also functional benefits such as UV resistance to protect against product degradation [22,23]. Moreover, pigments play a vital role in automotive paints, not only providing color but also enhancing durability, UV protection, and resistance to environmental conditions like corrosion (https://www.maximizemarketresearch.com/market-report/global-pigments-market/15119/, accessed on 28 November 2024).

## 3. Environmental Impact of Pigment Production

Natural pigments, often obtained through mining, pose significant environmental challenges. The extraction process causes severe ecosystems damage, depletes finite natural reserves, and leaves behind scarred landscapes, contributing to habitat destruction, deforestation, and biodiversity loss (https://earth.org/environmental-problems-caused-by-mining/; https://kunakair.com/environmental-impact-of-mining-in-air-quality/; access 3 November 2024), [25,26,27]. Mining can also contaminates local water sources through runoff containing heavy metals and harmful chemicals [28]. For example, copper extraction, commonly used to produce green and blue pigments, releases toxic and corrosive substances, such as sulfuric acid and heavy metals like lead and mercury into rivers and groundwater, posing risks to aquatic ecosystems and human health [29,30]. Additionally, mining is energy-intensive, requiring extensive fossil fuel use, which drives greenhouse gas emissions and exacerbates climate change.

Plant-based pigments may seem more eco-friendly, but they also have environmental drawbacks. Cultivating pigment-producing plants, such as indigo, demands large amounts of arable land and water, often resulting in deforestation, resource depletion, and unsustainable farming practices [26,31]. The use of synthetic fertilizers and pesticides in industrial-scale agriculture further pollutes the environment, harming soil quality, water bodies, and biodiversity. This raises concerns about the scalability and sustainability of plant-based pigment production.

Synthetic pigments, derived from petrochemicals, provide durability and cost efficiency but have had severe negative environmental and health implications. Many synthetic pigments contain heavy metals that leach into ecosystems, disrupting aquatic life through bioaccumulation and biomagnification [32,33,34]. Furthermore, synthetic pigments that are embedded in plastics degrade into microplastics, infiltrating marine systems and threatening both aquatic organisms and human health via the food chain [35]. The production of synthetic pigments generates hazardous air pollutants, including volatile organic compounds (VOCs) that contribute to air pollution and pose health risks to workers and nearby communities [36]. Due to their stability and non-biodegradable nature, synthetic pigments persist in the environment for extended periods, accumulating in soils, water systems, and living organisms, which makes them especially harmful.

In contrast to mining and agriculture, microorganisms such as algae, fungi, and bacteria (e.g., actinomycetes) offer a sustainable and environmentally friendly alternative for pigment production. These microorganisms can be cultivated in controlled environments, minimizing the ecological footprint associated with their growth and harvesting [37,38,39]. Moreover, microbial cultivation can occur on non-arable land or in bioreactors, further conserving valuable natural resources and avoiding competition with food crops. Their production process involves lower energy consumption and eliminates the need for harmful chemicals and solvents, making it less polluting and safer for workers and communities. Additionally, the use of marine-derived microorganisms supports water conservation, as these organisms can thrive in seawater. For example, algae can produce a variety of natural pigments, including carotenoids (orange and red) and chlorophyll (green), without requiring the vast land and water resources of traditional agriculture [40]. 

Microbial pigment production can also be scaled efficiently, offering a cost-effective solution that supports accurate and consistent pigment yields. Microorganisms provide a versatile platform for innovation, as genetic engineering can enhance pigment production, enabling the creation of customized colors and properties while adhering to green chemistry principles.

By embracing bio-based pigments derived from microorganisms, industries can meet the growing consumer demand for eco-friendly solutions while mitigating the ecological and societal costs of pigment production.

## 4. Toxicity, Safety, and Regulatory Frameworks for Pigments

Toxic pigments have long posed serious health risks. Lead-based whites (such as lead carbonate) were once popular in oil paints for their opacity and brightness, but lead is a powerful neurotoxin and is especially harmful to children, causing neurological damage, developmental delays, and organ failure with prolonged exposure [41,42]. As a result, its use has been heavily restricted. Similarly, cadmium reds (cadmium sulfide), known for their vivid color, are now largely phased out due to the dangers of cadmium, a carcinogen that can cause lung and kidney damage, especially when its dust or fumes are inhaled [43,44]. Arsenic-based greens, once used for their striking hues in paints and textiles, are also highly toxic. Arsenic can lead to acute poisoning and long-term health issues, including cancer; these pigments have been largely discontinued due to their serious health risks [45,46,47].

**Figure 2:**
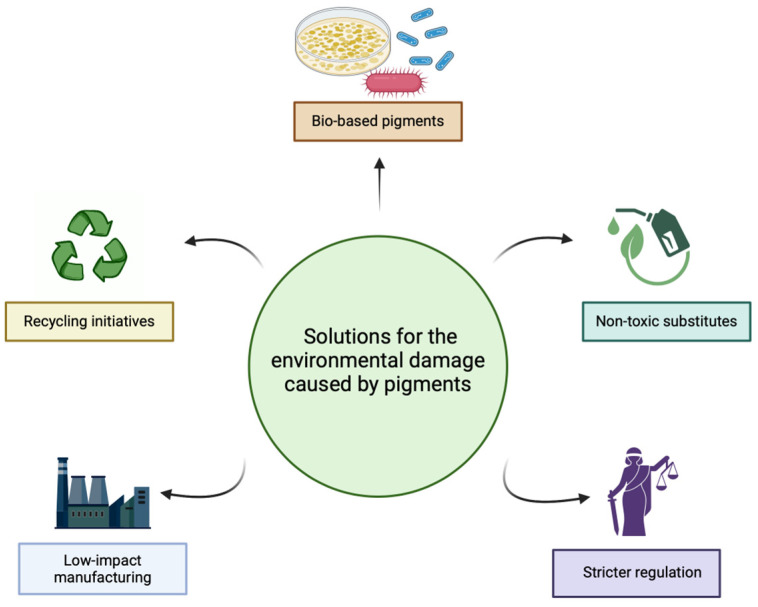
Pigment production solutions to mitigate environmental impacts. Created in https://BioRender.com.

Modern alternatives to toxic pigments have greatly improved both safety and performance. For example, titanium dioxide has largely replaced lead-based whites, offering a non-toxic option with excellent opacity and lightfastness, and it is safer to handle and more environmentally friendly [48]. Synthetic dyes have historically been associated with pollution, sparking increased interest in sustainable alternatives like biodegradable pigments derived from natural sources. While traditional pigments like lead, cadmium, and arsenic carry significant health risks, modern alternatives seek to offer non-toxic options without compromising color quality. Progress is being made in replacing toxic heavy metals (such as cadmium, lead, and chromium) with safer alternatives. Iron oxides, for instance, are commonly used as non-toxic replacements for red and yellow pigments. More recent advancements include the development of metal–organic frameworks (MOFs), which can replicate the properties of traditional pigments without the use of toxic metals [49,50,51]. Innovations to mitigate environmental concerns include bio-based pigments derived from microorganisms, non-toxic alternatives, recycling, low-impact manufacturing, and stricter regulations to reduce harmful chemical use and promote sustainability (Figure 2).

Eco-friendly manufacturing processes are being designed to lower energy consumption, eliminate harmful solvents, and reduce emissions. The adoption of green chemistry principles enables the creation of pigments in ways that minimize the release of toxic by-products.

Regulations and safety guidelines for pigments are enforced by various regulatory bodies. In the U.S., the Occupational Safety and Health Administration (OSHA; https://www.osha.gov/; accessed on 28 November 2024) and the Environmental Protection Agency (EPA; https://www.epa.gov/; accessed on 28 November 2024) regulate the handling and use of hazardous pigments. In the European Union, REACH (Registration, Evaluation, Authorisation, and Restriction of Chemicals; https://echa.europa.eu/regulations/reach/understanding-reach; accessed on 28 November 2024) imposes strict regulations on hazardous substances. These frameworks ensure safe handling and application, promoting health and environmental safety across industries. For food-grade pigments, regulatory bodies such as the Food and Drug Administration (FDA; https://www.fda.gov/food; accessed on 28 November 2024) in the U.S. and the European Food Safety Authority (EFSA; https://www.efsa.europa.eu/en; accessed on 28 November 2024) in Europe enforce strict safety standards.

Governments are also playing a crucial role by implementing stricter regulations on the use of hazardous pigments and the disposal of pigment-containing waste. For example, the European Union’s REACH regulations limit the use of certain toxic chemicals in pigments and encourage the development of safer alternatives.

Natural pigments are often preferred for their non-toxic properties and are marketed as healthier alternatives. These regulations ensure that any additives, including natural and synthetic pigments, are evaluated for safety and efficacy.

## 5. Pigments Produced by Actinomycetes

Actinomycete pigments come in a range of colors and have diverse applications across various industries. The most common examples include melanin (black/brown, produced by *Streptomyces glaucescens*) [52], carotenoids (yellow/orange, produced by *Streptomyces albidoflavus*) [53], prodigiosins (red, produced by *Streptomyces coelicolor*) [54], and actinorhodin (blue, also produced by *Streptomyces coelicolor*) [20,55]. Melanin, a dark pigment produced by several *Streptomyces* species, is highly valued for its protective properties against UV radiation and oxidative stress. Its stability and resistance to environmental degradation make it ideal for industrial use. Melanin’s durability and protective qualities have applications in cosmetics, particularly in sunscreens and anti-aging products. It is also used in textiles for natural black or brown dyes and in biomedicine for wound dressings and photoprotective treatments [52]. Carotenoids produce yellow, orange, and red hues, with *Streptomyces* species generating types such as lycopene, β-carotene, and astaxanthin. Known for their antioxidant properties, carotenoids are commonly used in the food industry as natural colorants and additives, offering non-toxic and health-boosting benefits. In cosmetics, they are added to skincare products for their ability to protect skin from oxidative damage. Carotenoids also hold value in the pharmaceutical industry due to their potential to combat diseases related to oxidative stress, such as cancer and cardiovascular issues [56,57]. Prodigiosins, red pigments produced by species like *Streptomyces coelicolor*, are recognized for their antimicrobial and anticancer properties [54]. These bioactive pigments show significant promise in the pharmaceutical industry as candidates for drug development, particularly for their potential in antimicrobial and anticancer therapies. In cosmetics, prodigiosins can be used to create natural red dyes with added health benefits. The textile industry also benefits from their use as a natural red dye, with the added functionality of producing antibacterial fabrics. Actinorhodin, a striking blue pigment produced by *Streptomyces coelicolor*, has both aesthetic and functional properties, including antimicrobial activity. It holds potential as a natural blue dye in the textile industry, offering a biodegradable and less toxic alternative to synthetic blue dyes. In the pharmaceutical industry, actinorhodin’s antimicrobial properties make it valuable for developing antibacterial treatments [58,59]. Additionally, its vibrant color and eco-friendly nature make it suitable for cosmetics, particularly in sustainable formulations.

A comprehensive search on the pigments produced by actinomycetes in the past 10 years is presented in Table 1. It showcases the diverse range of pigments produced by actinobacteria, highlighting not only their variety of colors, ranging from red and orange to blue, yellow, and black, but also their significant bioactivity, including antioxidant, antibacterial, antifungal, and antitumor effects.

The seventeen reported actinomycete-derived pigments exhibit wide chemical diversity and unique structural scaffolds; they belong to seven distinct chemical classes, including carotenoids, poliketides, prodiginines, antraquinones, flavonoids, melanins, and bis-indole alkaloids. These pigments showcase a range of bioactivities and applications, spanning various fields. Their potential uses include cosmetic formulations, therapeutic treatments, and other industrial applications, particularly in areas such as biofilm control, UV protection, and antimicrobial solutions.

Carotenoids, such as sioxanthin (**1**) (orange) and astaxanthin (**2**) (red), exhibit diverse bioactivities. Sioxanthin (**1**), produced by *Micromonospora* sp. SH-82, and *Salinispora tropica* (CNB-440), acts as a potent antioxidant. This pigment can be used in applications aimed at reducing oxidative stress, such as in skincare products and dietary supplements, helping to protect the skin from environmental damage and aging [60,61]. Similarly, astaxanthin (**2**), derived from *Corynebacterium glutamicum* and *Rhodococcus* sp., is widely recognized for its antioxidant capabilities. Although specific applications are not always detailed, antioxidants like astaxanthin are primarily used in cosmetics, skincare, and dietary supplements for protecting the skin against UV radiation, oxidative damage, and maintaining skin health [62,63].

The orange carotenoid sarcinaxanthin (**3**), produced by *Micrococcus luteus*, is notable for its antibacterial, antifungal, anticancer, and antioxidant properties [64]. These bioactivities suggest its potential use in treating infections, in cancer therapies, and as an antioxidant-rich ingredient in cosmetic products.

The red carotenoid bacterioruberin (**4**), derived from *Arthrobacter agilis* (NP20), also acts as an antioxidant and has applications in cosmetics, where it can protect the skin from environmental stressors like pollution and UV radiation [65].

The red ketocarotenoid all-trans-canthaxanthin (**5**), produced by *Gordonia jacobaea* (MV-1), has anti-UV and ROS-modulating effects [66]. Its primary applications include use in sunscreens and skincare products to protect against UV radiation and in food products as a natural pigment and antioxidant.

Other carotenoids include adonixanthin 3′-β-d-glucoside (**6**) from *Gordonia terrae* (TWRH01); it is an non-specific pigment in terms of bioactivity but is often studied for its antioxidant properties and potential applications in food and cosmetics [67].

Polyketides are represented by pigments such as tetracenomycin D (**7**) (blue), resistomycin (**8**) (orange), and resistoflavin (**9**) (yellow), all produced by *Streptomyces* sp. (EG1). These compounds are notable for their antibiofilm properties [68]. These pigments could be used in the development of treatments for biofilm-related infections or surfaces requiring biofilm inhibition.

Prodiginines like undecylprodigiosin (**10**), synthesized by *Streptomyces* sp. strains (SNA-077, BSE6.1, JAR6), demonstrate a wide range of bioactivities, including antitumor, immunosuppressant, antifungal, and antimalarial effects [69,70,71]. These properties suggest its potential application in cancer therapies, immune modulation, and as a treatment for fungal or malaria infections. Another member of this class, decylprodigiosin (**11**) and the related prodigiosin (red), produced by *Streptomyces violaceoruber* (CT-F61), are highlighted for their anticancer and antipathogenic activities, indicating their potential use in cancer treatments and as antimicrobial agents [72].

The red-orange anthraquinone 1-methoxy-3-methyl-8-hydroxy-anthraquinone (**12**) from *Actinomyces* sp. (AW6) shows antibacterial and antioxidant properties and also targets the Gyr-B enzyme [73]. Another member of this class is actinorhodin (**13**), which is produced by *Streptomyces lydicus* PM7 and *S. coelicolor* and has antibacterial activity [74,75]. These pigments could be applied in the development of antibacterial agents and antioxidants for pharmaceutical and personal care products.

Flavonoids such as the red-colored naringenin chalcone (**14**), produced by *Streptomyces clavuligerus*, and flavone (**15**) from *S. griseorubiginosus* exhibit antimicrobial and antioxidant bioactivities [76]. These pigments have potential applications in food preservation, as active ingredients in pharmaceutical formulations, and in cosmetics for their antioxidant and antimicrobial effects.

Melanins like pyomelanin (**16**) (dark brown), produced by multiple *Streptomyces* species (e.g., *Nocardiopsis* sp., *Streptomyces fuscus*, and *Actinoalloteichus cyanogriseus*), demonstrate cytotoxicity, antioxidant, antimicrobial, drug-binding, and anticancer properties [78,79,80,81,82]. These broad bioactivities make pyomelanin an attractive candidate for use in cancer therapies, antimicrobial agents, and anti-aging skincare products, where its protective properties against UV radiation and oxidative stress are highly valued.

Lastly, the blue-colored bis-indole alkaloid akashin A (**17**), produced by *Streptomyces* sp. (F001), lacks detailed bioactivity and application data, but similar pigments are often investigated for their potential antibacterial activity and therapeutic uses [83].

Actinomycete pigments offer a sustainable alternative to synthetic dyes, particularly in the textile industry. Natural pigments like actinorhodin, tetracenomycin D, akashin A (blue), prodigiosin, undecyl prodigiosin, bacterioruberin (red), and melanin (black/brown) provide biodegradable and environmentally friendly options that are far less harmful than petrochemical-based dyes. Carotenoids and other actinomycete pigments are increasingly used as natural food colorants, appealing to consumers seeking clean-label products with added health benefits. With the rising demand for natural, non-toxic cosmetics, pigments like melanin and carotenoids are commonly used in sunscreens, skincare products, and makeup, enhancing both the aesthetic and protective functionality of these products. Additionally, the bioactive pigments (Table 1) are promising for drug development, offering new possibilities for treating bacterial infections and cancer through their potent therapeutic properties.

## 6. Actinomycete Culture, Extraction Methods, and Challenges

The production of pigments from actinomycetes involves several techniques to culture and extract these valuable compounds. A comparative overview demonstrating the diversity in extraction methods, yields, and media optimization used to maximize pigment and bioactive compound production across various actinobacteria is presented in Table 2. Each strain has specific cultivation conditions and yields, emphasizing the role of media and extraction optimization in enhancing bioactive pigment yields from marine and terrestrial actinobacteria. The novel Actinobacterium *Micromonospora* sp. strain SH-82 was grown on A1 medium with starch, sea salts, yeast extract, and trace minerals, and was incubated at 28 °C for 10 weeks. After cultivation, metabolites were extracted through a sequential dichloromethane extraction process, and the solvents were evaporated under nitrogen. Although specific yield data were not provided, the extracts were prepared for high-performance liquid chromatography (HPLC) analysis [60]. *Streptomyces* sp. SNA-077 was cultured in 80 L of a starch-based medium at 27 °C with agitation for 168 h. Its active metabolites were then extracted with chloroform using medium-pressure liquid chromatography (MPLC), and further purified by thin-layer chromatography (TLC) and reversed-phase HPLC, yielding 7.8 mg of the pigment undecylprodigiosin [69]. *Streptomyces* sp. EG1 was first grown on Waksman agar and later transferred to liquid media, where it was fermented at 28 °C for seven days. Both the broth and mycelia were extracted using ethyl acetate and methanol, resulting in a 3.67 g crude extract. This included significant yields of bioactive compounds, particularly 10 mg of tetracenomycin D, 25 mg of resistomycin, and 5 mg of resistoflavin [68]. *Arthrobacter agilis* NP20 was cultivated in a PY medium and an alternative cheese-whey-based medium, optimized for carotenoid pigment production. Extraction involved bleaching the cells with methanol to retrieve pigments, achieving a yield of 5.13 mg/L of carotenoids [65]. *Streptomyces tunisiensis* W4MT573222 exhibited a 12.2-fold increase in pigment yield in an optimized starch-based medium. Pigment extraction was conducted using a 7:3 acetone and methanol mixture [84]. *Streptomyces* sp. LS1 was cultured in a Luria–Bertani Modified Medium (LB) at 30 °C with shaking over a seven-day period. Pigments were sequentially extracted from both the extracellular and intracellular sources using ethanol, yielding 30 mg of purified pigment [85]. *Streptomyces maritimus* AJ6 and *Streptomyces fenghuangensis* AJ7 were grown on specialized media and screened for pigment production. Ultrasonication followed by liquid–liquid methanol extraction retrieved intracellular pigments with impressive yields of 12.9 g/L and 13.8 g/L, respectively [86]. *Actinomyces* sp. AW6 was cultivated on starch nitrate agar, and bioactive metabolites were extracted using ethyl acetate, yielding 13 g of dry extract [73]. *Streptomyces enissocaesilis* SSASC10 was grown on starch casein agar, which encouraged the production of pigmented colonies. The pigments were extracted with a series of solvents, including methanol, chloroform, ethyl acetate, and n-hexane [87]. *Gordonia terrae* TWRH01 was cultured in a PYC medium containing yeast extract, peptone, glucose, and calcium chloride at 30 °C over seven days. Carotenoid pigments were extracted via sonication in acetone, achieving a dry biomass yield of 5.29 g/L and a β-carotene concentration of 10.58 μmol/L [67].

Among the listed microorganisms, the conditions yielding the best pigment production in terms of concentration and yield were observed for *Streptomyces maritimus* AJ6 and *Streptomyces fenghuangensis* AJ7, which produced 12.9 g/L and 13.8 g/L of intracellular pigments, respectively. Both strains were cultivated on specialized media, with pigments extracted through ultrasonication followed by liquid–liquid extraction using methanol [86]. Another significant yield was achieved by *Actinomyces* sp. AW6, which produced 13 g of dry extract after being cultured on starch nitrate agar, although specific pigment yield data were not provided [73]. These findings suggest that *Streptomyces maritimus* AJ6 and *Streptomyces fenghuangensis* AJ7 achieved the highest pigment production, with the specialized media and ultrasonication-based extraction method yielding especially high concentrations.

**Figure 3:**
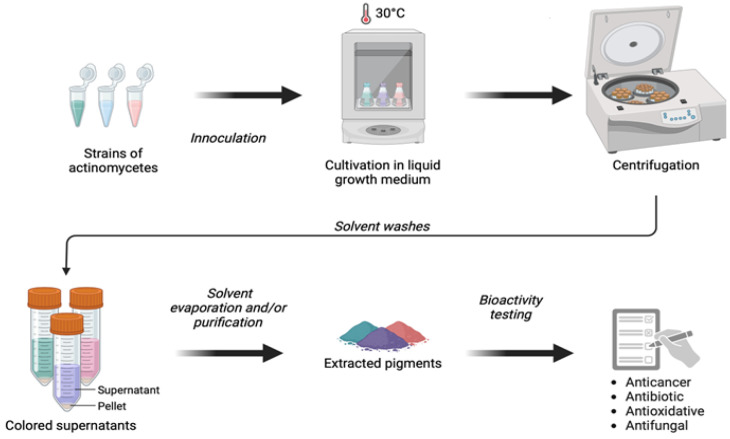
Culture and extraction of bacterial pigments. Created in https://BioRender.com.

The two key stages in the actinomycete pigment production process are fermentation and extraction (Figure 3). Actinomycetes are typically cultured using fermentation methods, where the bacteria grow in liquid (submerged fermentation, SmF) or solid (solid-state fermentation, SSF) media under controlled conditions. SmF is the most common approach, where actinomycetes are grown in a nutrient-rich broth. This method allows for the optimal control of environmental factors such as temperature, pH, and oxygen levels, which are crucial for maximizing pigment production. Another approach, SSF, involves growing actinomycetes on solid substrates, like agricultural waste. This method mimics the natural soil conditions in which many actinomycetes thrive and can reduce production costs by utilizing inexpensive raw materials.

Once pigments are produced, they must be extracted from the bacterial culture. The most common method is solvent extraction, which involves using organic solvents like methanol, acetone, or ethyl acetate to dissolve the pigments from the bacterial cells or fermentation medium. After extraction, the solvent is evaporated, leaving behind the pigments, which may require further purification steps. Other methods, such as ultrasound-assisted extraction and supercritical fluid extraction, are emerging as more efficient and eco-friendly alternatives, although these techniques are still under development for large-scale use. Each method has its advantages and drawbacks, depending on factors like yield, purity, and cost.

Optimizing cultivation conditions, such as the medium, temperature, incubation time, pH, and agitation and aeration conditions, along with selecting appropriate extraction conditions, including the solvent and method, is essential for maximizing the yield of actinomycete-derived pigments. These factors must be carefully considered for each species to achieve the desired outcomes when pursuing biotechnological applications. The pigment produced is influenced by the species of actinomycetes, as different strains possess distinct metabolic pathways and capabilities. These processes are influenced not only by the species but also by the composition of the cultivation medium, as variations in media can influence their metabolism (carbon source, nitrogen source, and C/N ratio). The use of specialized media or additives like calcium chloride, magnesium sulfate, or iron sulfate are often employed to enhance pigment production. For example, the culturing of *Streptomyces albidoflavus* and *Actinomadura* sp. BRA 177 uses specific supplementation to the media to improve the yield of pigments [53,89]. Additionally, the polarity of the extraction solvent can affect which pigments are extracted, as different solvents selectively dissolve pigments with varying polarities. For instance, methanol was utilized for cell extraction from *Arthrobacter agilis* NP20 to obtain bacterioruberin [65], while acetone extraction with sonication was used for *Gordonia terrae* TWRH01 to obtain β-carotene, producing a yield of 10.58 μmol/L [67]. The choice of solvent and extraction method directly impacts the amount of pigment obtained from the culture, as was seen with *Streptomyces* sp. EG1, where 10 mg of tetracenomycin D was obtained using ethyl acetate extraction [68].

The OSMAC (One Strain, Many Compounds) technique is valuable in this context as it allows for the evaluation of the best conditions for pigment production by altering various parameters such as medium composition, temperature, aeration conditions, and extraction solvent, among others [90]. By systematically testing different conditions, OSMAC can help identify the optimal environment for producing specific pigments, improving both yield and bioactivity [91,92]. Moreover, the Response Surface Methodology (RSM) is a powerful tool for analyzing multivariate data derived from well-designed experiments. It facilitates the optimization of culture media, process parameters, and extraction conditions to enhance pigment production [93,94]. 

## 7. Scaling up Pigment Production 

The scaling up of actinomycete pigments for industrial applications has advanced significantly through genetic engineering, fermentation optimization, and cost-effective strategies. Furthermore, the upcycling process offers an innovative approach to reduce production costs by efficiently utilizing raw materials, energy, and labor while maintaining consistent pigment yields under optimized controlled environments [95,96,97]. This aligns with the circular economy principles by promoting resource efficiency, minimizing waste, and encouraging the sustainable use of materials. Furthermore, this strategy supports the UN Sustainable Development Goals (SDGs), particularly Goal 9 (Industry, Innovation, and Infrastructure), Goal 12 (Responsible Consumption and Production), Goal 13 (Climate Action), and in case of marine-derived actinomycetes, Goal 14 (Life Below the Water).

Genetic engineering has the power to revolutionize the field of pigment production by enabling targeted modifications in actinomycetes and facilitating the transfer of complex biosynthetic pathways to heterologous hosts. These advances not only enhance the efficiency and scalability of pigment production but also open avenues for the creation of novel pigments with tailored properties. As tools like CRISPR, synthetic biology, and metabolic engineering continue to evolve, the potential for innovation in this field remains immense.

One approach to enhancing pigment production in actinomycetes involves the activation and overexpression of biosynthetic pathways. Many pigments are synthesized by gene clusters that are either silent or expressed at low levels under normal conditions. To activate these pathways, native promoters can be replaced by stronger, constitutive, or inducible ones. Additionally, amplifying the copy number of biosynthetic genes using plasmid-based systems or genome-editing techniques, such as CRISPR/Cas9, can provide higher enzyme availability, leading to an increased metabolic flux through pigment synthesis pathways [98,99,100,101].

Metabolic engineering further supports pigment production by redirecting resources within the cell. For example, knocking out competing metabolic pathways or enhancing the biosynthesis of precursor molecules increases the efficiency of pigment production. Technologies such as CRISPR interference (CRISPRi) and antisense RNA can downregulate non-essential pathways, while overexpression of key enzymes ensures a steady supply of precursors [102,103,104]. Modifications to regulatory systems, including the deletion of global repressors or the overexpression of pathway-specific activators, have also proven to be effective in boosting yields.

Prominent examples of this approach involve the enhancement of prodigiosin production in *Streptomyces* strains [105]. One effective approach involves combinatorial metabolic engineering to significantly increase prodigiosin production. In *Streptomyces coelicolor*, this method includes inactivating the repressor gene ohkA, deleting competing antibiotic biosynthetic gene clusters, and integrating multiple copies of the prodigiosin biosynthetic gene cluster into the chromosome [54]. Another strategy utilizes collagen peptides to provide abundant precursors for the production of undecylprodigiosin, a prodigiosin analog, in *Streptomyces coelicolor* [106]. This method has resulted in enhanced pigment yields, demonstrating the importance of precursor availability in prodigiosin biosynthesis. Additionally, the use of heat-killed cells of *Lactobacillus rhamnosus* has been shown to enhance prodigiosin production in *Streptomyces coelicolor*, indicating that certain microbial interactions or components can stimulate pigment biosynthesis [107]. Moreover, undecylprodigiosin was produced in high yields by *Streptomyces* sp. ALAA-R20, a strain developed through protoplast fusion of marine endophytic strains [108]. Solid-state fermentation using groundnut oil cake and wastewater yielded 181.78 mg/gds, providing a cost-effective approach for large-scale production [108]. While native actinomycete strains are excellent sources of pigments, their complex growth requirements and genetic regulation often present challenges for industrial-scale production. To address these issues, heterologous expression is used, where pigment biosynthetic pathways are transferred into alternative hosts, such as *Escherichia coli* or *Saccharomyces cerevisiae* [109]. These hosts are genetically simpler and grow rapidly, making them well-suited for large-scale production.

The advantages of heterologous hosts include scalability, simplified genetic systems, and the ability to modify biosynthetic pathways to produce novel pigment derivatives. This strategy has been used successfully in numerous examples, such as the production of anthraquinones in *E. coli* by transferring pathways from *Streptomyces* and the synthesis of carotenoids in yeast for food and cosmetic applications [110]. Additionally, the use of waste substrates for carotenoid-rich yeast biomass production demonstrated the feasibility of utilizing agro-industrial wastes to produce high levels of carotenoids using yeast [111]. This approach not only provides a cost-effective method for carotenoid production but also contributes to waste valorization.

Other scale-up examples include indigoidine production in *Streptomyces lividans* TK24. It was enhanced using a refined self-regulation system and optimized σ^hrdB expression, coupled with the use of industrial glycerol as a low-cost carbon source [112]. This approach achieved yields of 14.3 g/L in flasks and 46.27 g/L in 4 L fermenters, the highest microbial indigoidine production reported to date [112]. Similarly, *Streptomyces chromofuscus* ATCC 49982 produced 14 g/L of indigoidine in a 5 L bioreactor by using engineered protein scaffold complexes to improve enzymatic activity and metabolic efficiency [113].

Astaxanthin production in *Corynebacterium glutamicum* was significantly enhanced through pathway engineering and optimization of the pH and fermentation parameters [114]. Fed-batch cultivation yielded 64 mg/L, while genetic modification increased β-carotene production by 1.5-fold and astaxanthin production increased nearly 5- fold, achieving a peak yield of 103 mg/L in a 2 L fermenter [114]. Furthermore, the co-production of amino acids and carotenoids in *C. glutamicum* demonstrated scalability, with engineered strains producing 48 g/L of L-lysine and 10 mg/L of astaxanthin in a 20 L bioreactor, using alternative feedstocks like xylose and arabinose, supporting sustainable processes [62].

Neopurpuratin production using *Streptomyces propurpuratus* IX-30 was scaled up though mutagenesis and the use of cost-effective media, such as soybean meal, achieving a yield increase of more than 2-fold to over 5000 µg/mL [115]. When scaling up fermentation from 30 L to 1500 L, a high product quality was maintained, with over 93% purity, demonstrating industrial feasibility [115].

Phycocyanobilin (PCB), a valuable antioxidant, was produced in *Corynebacterium glutamicum* by enhancing the heme biosynthesis and pentose phosphate pathways to maximizing NADPH generation [116]. This process achieved 259.63 mg/L in a 5 L bioreactor, representing the highest bacterial PCB yield reported and offering a sustainable alternative to algal extraction [116].

Canthaxanthin production was optimized in *Dietzia natronolimnaea* HS-1 and *Brevibacterium* sp. KY-4313 [117,118]. Mutagenesis increased the yields by 32%, while media optimization using fumaric acid–molasses supported superior growth and a pigment production of 9.3 mg/L [117]. Continuous culture with enzymatic hydrolysate molasses (EHM) achieved a yield of 25.04 mg/L canthaxanthin in a 2.5 L bioreactor, showcasing the effective use of waste-derived substrates [118].

Identifying scalable, renewable, and cost-effective substrates is crucial for making actinomycete pigments economically competitive with synthetic alternatives. Utilizing these substrates reduces dependence on conventional media, lowering production costs and promoting sustainability. Agricultural waste and other low-cost materials can serve as effective substrates, and maintaining cost efficiency at an industrial scale will greatly benefit from continued research and optimization. Additionally, marine-derived actinomycetes offer the advantage of incorporating seawater into the media composition, significantly reducing the demand for freshwater resources.

The extraction and purification of pigments have seen significant advancements. While solvent extraction is a widely used method, it often requires large quantities of organic solvents, presenting cost and environmental challenges. However, innovative techniques like supercritical fluid extraction offer more efficient and environmentally friendly alternatives. With ongoing research and development, these newer methods hold the potential to become more cost-effective and scalable, paving the way for sustainable pigment production.

Despite industrial development challenges, actinomycete-based pigments offer significant environmental benefits. They are renewable, biodegradable, and non-toxic, aligning with the growing demand for sustainable alternatives to synthetic dyes. As more companies focus on reducing their environmental impact, there may be increasing interest in investing in natural pigment production, even if the initial costs are higher. The feasibility of large-scale production could improve with advancements in biotechnology. Innovations in genetic engineering, metabolic pathway optimization, and bioprocessing techniques may help increase pigment yields and reduce production costs [95,96]. Additionally, the development of more efficient extraction and purification technologies could make actinomycete pigments more commercially viable in the future. The demonstrated commercial viability and bioactive properties of actinomycete pigments, alongside reduced costs and environmental impact, highlight their potential to revolutionize industries like the nutraceutical, cosmetic, and food industries, while advancing global sustainability goals.

## 8. Economic Viability and Market Potential of Actinomycete Pigment Production

Producing pigments from actinomycetes offers promising economic opportunities, especially when considering the growing demand for sustainable alternatives. While synthetic pigments currently benefit from cost-effectiveness due to established petrochemical processes, efficient supply chains, and economies of scale, actinomycete pigment production has distinct advantages [24,119,120,121]. Although the initial costs associated with fermentation technology and purification methods may be high, these expenses are offset by the increasing consumer preference for eco-friendly products. Moreover, microbial pigment production, including actinomycetes, can be efficiently scaled, ensuring accurate and consistent yields while paving the way for cost-effective solutions. As biotechnological advancements continue to improve production methods, the cost of actinomycete pigments is expected to decrease, further enhancing their economic viability and making them a competitive alternative to synthetic pigments.

Consumer trends are increasingly favoring green products, driven by the growing awareness of environmental issues and health concerns associated with synthetic chemicals [121]. This shift has led to a rising demand for sustainably sourced pigments across various industries, including the textile, food, cosmetic, and pharmaceutical industries. Consumers are seeking products that align with their values, preferring natural ingredients that are safe, biodegradable, and environmentally friendly. Market research indicates a strong growth trajectory for natural and organic products. For instance, the global natural food colorant market is projected to expand significantly as more consumers opt for natural alternatives to synthetic dyes (https://www.precedenceresearch.com/natural-food-color-market; accessed on 25 September 2024). Similarly, the cosmetic industry is witnessing a surge in demand for clean beauty products, with consumers increasingly scrutinizing ingredient lists and favoring brands that prioritize sustainability [24]. As such, actinomycete-derived pigments stand to benefit from these market trends, positioning themselves as viable alternatives to both synthetic and traditional natural pigments. Companies that adopt these eco-friendly pigments can enhance their brand image, attract environmentally conscious consumers, and potentially command premium pricing for their products.

Navigating regulatory frameworks can pose significant challenges, particularly in industries like the food and pharmaceutical industries, where new natural pigments must meet strict safety and efficacy standards before being approved for widespread commercial use. Actinomycete-derived pigments are subject to extensive testing and documentation to comply with the approval processes of regulatory bodies such as the FDA and EFSA. To effectively address these challenges, stakeholders must conduct comprehensive research and development, ensure compliance with safety standards, and educate regulatory bodies on the advantages and safety of actinomycete pigments.

Although there is growing interest in sustainable products, consumer awareness of actinomycete-derived pigments remains limited. Educating consumers and industry stakeholders about the benefits and functionalities of these pigments is essential for driving adoption. Despite these challenges, considerable opportunities exist for the commercialization of actinomycete pigments. Collaborations between academic institutions, industry stakeholders, and biotechnology firms can foster innovation and accelerate the sustainable production of these pigments [122,123].

Actinomycete pigments also have the potential to find a strong foothold in niche markets that prioritize sustainability, such as organic textiles, health-conscious food products, and eco-friendly cosmetics. Targeting these markets can significantly enhance the appeal of actinomycete pigments. Additionally, exploring new applications for these pigments, such as in bioplastics or as natural preservatives, can open additional revenue streams and further expand their market potential.

## 9. Case Studies and Examples of Industrial Use of Actinomycetes-Derived Pigments

Actinomycete-derived pigments are gradually gaining traction in various industries, reflecting a growing trend toward sustainable and eco-friendly alternatives to synthetic dyes. BOC Sciences showcased the successful production and commercialization of these pigments, demonstrating their potential to enhance sustainability and reduce environmental impact.

BOC Sciences (https://bio-fermen.bocsci.com/ accessed on 12 December 2024), a global supplier of biochemicals and specialty compounds, has made significant strides in developing and commercializing actinomycete-derived pigments. By focusing on biotechnological advancements, the company offers a wide array of natural pigments. Their expertise in fermentation and downstream processing ensures that actinomycete-derived pigments can be produced cost-effectively and at scale, making them accessible for industrial use. This company uses several *Streptomyces* and *Nocardiopsis* species to produce pigments such as astaxanthin (https://bio-fermen.bocsci.com/fermentation-products.html; accessed on 12 December 2024).

BOC Sciences has successfully commercialized several actinomycete-derived pigments, demonstrating their industrial scalability and sustainability. One example is rhodomycin, a red pigment with antibiotic and antifungal activities, produced by a mutant strain of *Streptomyces griseus*. This pigment shows great promise for medical applications. Another example is actinorhodin, a blue pigment with antibacterial and antiviral (HIV) properties that is produced by *Streptomyces coelicolor*. Melanin, a black pigment produced by *Streptomyces griseus*, is valued for its UV-protective properties, particularly in the cosmetic industry. In addition, resistoflavin, which is produced by several *Streptomyces* species, has found applications in cancer research.

While actinomycetes are a primary source of natural pigments, other microorganisms are also used by BOC Sciences in pigment production. *Phaffia rhodozyma*, a yeast, produces carotenoids, including astaxanthin, which is used in applications such as health products, medicines, cosmetics, food additives, and in aquaculture. *Serratia* species produce the red pigment prodigiosin, which exhibits antifungal, anticancer, and antimalarial activities. In addition to its medical potential, prodigiosin is well-suited for use in cosmetics, textiles, and as a food colorant.

These pigments are not only valued for their color but also for their bioactivity, which makes them useful in various biotechnological applications. In pharmaceuticals, they serve as antibiotics, anticancer agents, and immunosuppressants. In cosmetics, pigments like astaxanthin and melanin are used for their antioxidant properties and to enhance skin health. In the food and nutraceutical industries, carotenoids such as astaxanthin and β-carotene serve as natural colorants and dietary supplements.

Actinomycete-derived pigments have successfully transitioned from laboratory research to commercial applications, gaining attention for their eco-friendly, sustainable, and versatile properties. Companies like BOC Sciences exemplify this success by effectively leveraging fermentation technologies to produce and market these natural pigments. Their efforts demonstrate the feasibility of scaling up production while maintaining cost efficiency and environmental benefits. The demonstrated industrial scalability and eco-conscious production methods highlight the growing commercial success of actinomycete-derived pigments.

## 10. Conclusions and Future Prospects

The future of actinomycete pigments is highly promising, and is driven by ongoing research and innovations in biotechnology and genetic engineering aimed at enhancing their yield, stability, and functionality. Techniques like CRISPR and metabolic engineering are being employed to modify actinomycetes’ metabolic pathways, boosting pigment production and enabling the development of new pigments with advanced properties, such as antioxidant and antimicrobial activities. These efforts are complemented by optimized fermentation processes, such as fed-batch fermentation, to maximize yield and maintain stability.

The use of actinomycete pigments aligns closely with the growing demand for natural, biodegradable, and non-toxic solutions in industries like the food, cosmetic, textile, and pharmaceutical industries. Their unique properties, including UV protection, antioxidant benefits, and anticancer effects, position them as eco-friendly alternatives to synthetic dyes. By reducing the need for chemical preservatives and reducing the environmental impacts, these pigments with antimicrobial and antioxidant properties also support circular economy principles.

The global shift toward sustainability, clean-label products, and stricter regulations on synthetic dyes further drives the adoption of actinomycete-derived pigments. Although scaling up production presents challenges in terms of cost-effectiveness and technology, the environmental benefits, such as reduced chemical waste, the use of seawater and industrial wastes for fermentation, a smaller carbon footprint, and fewer resource demands, make them highly viable for sustainable manufacturing. As the focus on circular economies and sustainability increases, actinomycete pigments will be pivotal in greener biotechnological applications.

Actinomycete pigments hold a significant potential to meet the rising demand for greener alternatives. Their market potential is bolstered by increasing consumer preferences for sustainable products and collaborative research efforts, positioning actinomycete pigments as key contributors to more sustainable industrial practices. Advances in genetic engineering, fermentation techniques, and the use of cost-effective substrates have significantly enhanced production yields while aligning with circular economy principles and the UN Sustainable Development Goals. For instance, microbial fermentation processes have been optimized for pigments like indigoidine, astaxanthin, and undecylprodigiosin. Innovations such as self-regulating systems, alternative carbon sources like glycerol, and engineered protein scaffolds have led to unprecedented yields in lab and pilot-scale bioreactors. The use of agricultural waste, seawater-based media, and low-cost feedstocks further enhances the sustainability and cost efficiency of their production.

Actinomycete-derived pigments have transitioned successfully from lab to commercial markets, and are recognized for their sustainability, eco-friendliness, and adaptability in various industries. Companies like BOC Sciences (Shirley, NY, USA) lead this trend, utilizing advanced fermentation technologies to produce scalable, cost-efficient, and environmentally conscious natural pigments. These innovations highlight the growing commercial success and viability of actinomycete-based pigments in replacing synthetic alternatives.

## Figures and Tables

**Table 1:** Actinomycete species and the pigments they produce, along with their associated bioactivities and potential applications. N.R.—not reported.

Chemical Class	Species	Pigment Name	Chemical Structure	Bioactivity Application	Ref(s).
Carotenoid	*Micromonospora* sp. SH-82, *Salinispora tropica* CNB-440	Sioxanthin (orange)	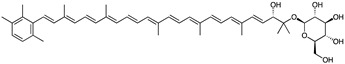 **1**	Antioxidant	[60,61]
	*Corynebacterium glutamicum*, *Rhodococcus* sp.	Astaxanthin (red)	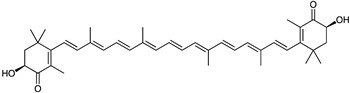 **2**	N.R.	[62,63]
	*Micrococcus luteus*	Sarcinaxanthin (orange)	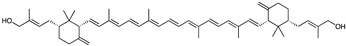 **3**	Antibacterial Antifungal Anticancer Antioxidant	[64]
	*Arthrobacter agilis* NP20	Bacterioruberin (red)	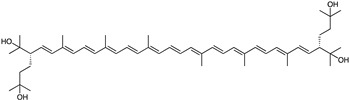 **4**	Antioxidant Cosmetics	[65]
	*Gordonia jacobaea MV-1*	Ketocarotenoid all-*trans*-canthaxanthin (4,4′-diketo-β-carotene) (red)	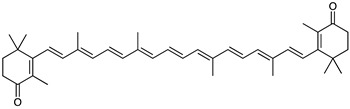 **5**	Anti-UV radiation ROS membrane fluidity modulator	[66]
	*Gordonia terrae* TWRH01	Adonixanthin 3′-β-d-glucoside (orange-red)	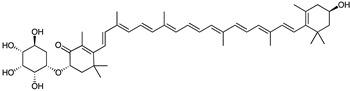 **6**	N.R.	[67]
Polyketide	*Streptomyces* sp. *EG1*	Tetracenomycin D (blue) Resistomycin (orange) Resistoflavin (yellow)	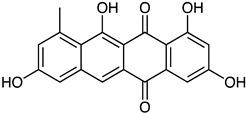 **7** 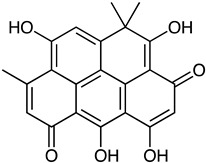 **8** 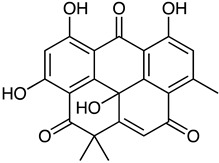 **9**	Antibiofilm	[68]
Prodiginine	*Streptomyces* sp. SNA-077, *Streptomyces* sp. BSE6.1, *Streptomyces* sp. JAR6	Undecylprodigiosin (red)	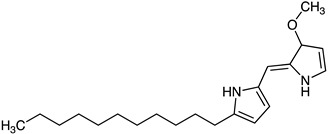 **10**	Antitumor Immunosuppressor Antifungal Antimalarial	[69,70,71]
	*Streptomyces violaceoruber CT-F61*	Decylprodigiosin (red)	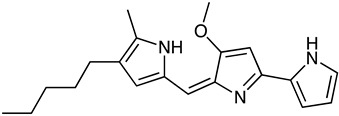 **11**	Anticancer Antipathogenic	[72]
Anthraquinone	*Actinomyces* sp. AW6	1-methoxy-3-methyl-8-hydroxy-anthraquinone (red-orange)	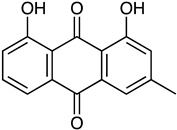 **12**	Antibacterial Antioxidant Anti-Gyr-B enzyme	[73]
	*Streptomyces lydicus PM7*, *Streptomyces coelicolor*	Actinorhodin (red-blue)	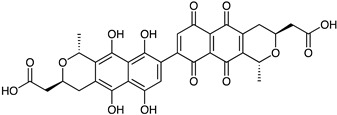 **13**	Antibacterial	[74,75]
Flavonoid	*Streptomyces clavuligerus*	Naringenin chalcone (red-brown)	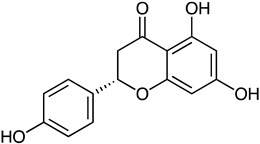 **14**	N.R.	[76]
	*S. griseorubiginosus*	Flavone (red)	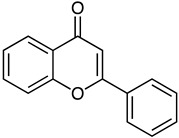 **15**	Antimicrobial Antioxidant	[77]
Melanin	*Nocardiopsis* sp., *Streptomyces fuscus* sp. *GXMU- J15T*, *Streptomyces* sp. *MR28*, *Actinoalloteichus cyanogriseus*, *Streptomyces djakartensis NSS-3*	Pyomelanin (dark brown)	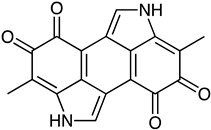 **16**	Cytotoxicity Antioxidant Antimicrobial Drug binding Anticancer	[78,79,80,81,82]
Bis-Indole Alkaloid	*Streptomyces* sp. *F001*	Akashin A (blue)	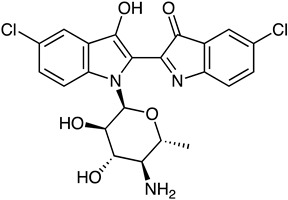 **17**	N.R.	[83]

**Table 2:** Actinomycetes pigment fermentation production and extraction methods.

Species	Culture Conditions (1L)	Extraction Conditions	Yield	Reference
*Actinobacterium Micromonospora* sp. nov. *SH-82*	A1 medium composition: 10 g soluble starch, 33 g sea salts, 4 g yeast extract Supplementation: 1 g calcium carbonate, 2 g peptone, 100 mg potassium bromide, 40 mg ferric sulfate Incubation: 28 °C, 10 weeks	CH_2_Cl_2_ extraction	Sioxanthin	[60]
*Streptomyces* sp. SNA-077	10 g soluble starch, 2 g yeast extract, 4 g peptone, 34.75 g NaCl Incubation: 120 rpm, 27 °C, 7 days	CHCl_3_ phase separation Reverse-phase HPLC isolation	7.8 mg undecylprodigiosin	[69]
*Streptomyces* sp. *EG1*	Waksman agar medium: 20 g glucose, 5 g meat extract, 5 g peptone, 3 g dried yeast, 5 g NaCl, 3 g calcium carbonate (CaCO_3_), and 15 g agar Incubation: 200 rpm, 28 °C, 7 days	Ethyl acetate extraction	10 mg tetracenomycin D 25 mg resistomycin 5 mg resistoflavin	[68]
*Arthrobacter agilis* NP20	PY: 10 g peptone, 5 g yeast extract, 5 g NaCl Cheese-whey-based medium: 960 g sweet whey, 4.6 g magnesium sulfate (MgSO_4_), 5.0 g yeast extract Incubation: 25 °C, 72 h	Methanol cell extraction	5.13 mg bacterioruberin	[65]
*Streptomyces tunisiensis* W4MT573222	5 g starch, 0.88 g potassium nitrate (KNO_3_), 1 g dipotassium phosphate (K_2_HPO_4_), 0.025 g magnesium sulfate (MgSO_4_), 0.015 g ferrous sulfate (FeSO_4_), 0.65 g casein, 0.03 g calcium carbonate (CaCO_3_)	Acetone/methanol (7:3) extraction	12.2-fold increase in pigment production	[84]
*Streptomyces* sp. LS1	Luria–Bertani Modified Medium (LB; 50 mL): 10 g starch, 2 g peptone, 4 g yeast extract Incubation: 200 rpm, 30 °C, 7 days	Ethanol extraction with sonication	30 mg carotenoids	[85]
*Streptomyces maritimus* AJ6 *Streptomyces fenghuangensis* AJ7	ISP7 medium	Methanol extraction with sonication	12.9 and 13.8 g actinomycin	[86]
*Actinomyces* sp. AW6	20 g starch, 0.5 g dipotassium phosphate (K_2_HPO_4_), 1 g potassium nitrate (KNO_3_), 0.5 g magnesium sulfate heptahydrate (MgSO_4_·7H_2_O), 0.01 g ferrous sulfate (FeSO_4_), 50% from a marine source Incubation: 30 °C, 7 days	Ethyl acetate extraction	13 g anthraquinone	[73]
*Streptomyces enissocaesilis* SSASC10	Soybean casein digest broth Incubation: 80 rpm, 37 °C, 5–7 days	Methanol 1:2 CHCl_3_/H_2_O, 1:2 ethyl acetate/H_2_O 1:2 n-hexane/H_2_O extraction	_	[87]
*Gordonia terrae TWRH01*	Peptone–Yeast Extract–Calcium Chloride (PYC) medium: 1 g yeast extract, 15 g peptone, 20 g glucose, 0.45% (*v*/*v*) 1 M calcium chloride (CaCl_2_) solution Incubation: 150 rpm, 30 °C, 7 days	Acetone extraction with sonication	5.29 g L^−1^ dry biomass 10.58 μmol L^−1^β-carotene	[67]
*Streptomyces* sp. *BSE6.1*	Marine agar Other media: nutrient agar, luminescent agar, Photobacterium agar, *Pseudomonas* fluorescence agar, *Pseudomonas* pyocyanin agar, *Bacillus* agar, seawater agar, casitose agar Incubation: 32 °C, 7–10 days	Methanol extraction	Undecylprodigiosin	[70]
*Actinomycete RA2* isolated from the Red Sea sponge *Spheciospongia mastoidea*	Waksman agar medium: 20 g glucose, 5 g meat extract, 5 g peptone, 3 g dried yeast, 5 g sodium chloride (NaCl), 3 g calcium carbonate (CaCO_3_), 1.5 g agar Incubation: 28 °C, 15 days	Methanol extraction	2.6 g of prodigiosins	[88]
*Streptomyces albidoflavus*	Casein agar (SCA): soluble starch (1%), casein (0.03%), salts: potassium nitrate (KNO_3_), magnesium sulfate heptahydrate (MgSO_4_·7H_2_O), dipotassium phosphate (K_2_HPO_4_), sodium chloride (NaCl), calcium carbonate (CaCO_3_), ferrous sulfate heptahydrate (FeSO_4_·7H_2_O)) Incubation: 30 °C, 7–15 days	Methanol extraction	1.0866 ± 0.0089 mg% 2.5 g/l flexirubin	[53]
*Actinomadura* sp. *BRA 177*	A1 medium Supplementation: calcium carbonate (CaCO_3_), iron(III) sulfate (Fe_2_(SO_4_)_3_), potassium bromide (KBr) Incubation: 150 rpm, 28 °C, 10 days	Ethyl acetate extraction	390.8 mg nonylprodigiosin, cyclononylprodigiosin, and methylcyclooctilprodigiosin	[89]
